# Airway and Lung Organoids from Human-Induced Pluripotent Stem Cells Can Be Used to Assess CFTR Conductance

**DOI:** 10.3390/ijms24076293

**Published:** 2023-03-27

**Authors:** Anna Demchenko, Ekaterina Kondrateva, Vyacheslav Tabakov, Anna Efremova, Diana Salikhova, Tatiana Bukharova, Dmitry Goldshtein, Maxim Balyasin, Natalia Bulatenko, Elena Amelina, Alexander Lavrov, Svetlana Smirnikhina

**Affiliations:** 1 Laboratory of Genome Editing, Research Centre for Medical Genetics, Moskvorechye, 1, 115522 Moscow, Russia; 2 Moscow Branch of the Biobank “All-Russian Collection of Biological Samples of Hereditary Diseases”, Research Centre for Medical Genetics, Moskvorechye, 1, 115522 Moscow, Russia; 3 Stem Cell Genetics Laboratory, Research Centre for Medical Genetics, Moskvorechye, 1, 115522 Moscow, Russia; 4 Scientific and Educational Resource Center, Peoples’ Friendship University of Russia, Miklukho-Maklaya, 6, 117198 Moscow, Russia; 5 Laboratory of Cystic Fibrosis, Research Institute of Pulmonology, 11th Parkovaya Str., 32/4, 105077 Moscow, Russia

**Keywords:** human-induced pluripotent stem cells, differentiation, organoids, cystic fibrosis, CFTR

## Abstract

Airway and lung organoids derived from human-induced pluripotent stem cells (hiPSCs) are current models for personalized drug screening, cell–cell interaction studies, and lung disease research. We analyzed the existing differentiation protocols and identified the optimal conditions for obtaining organoids. In this article, we describe a step-by-step protocol for differentiating hiPSCs into airway and lung organoids. We obtained airway and lung organoids from a healthy donor and from five donors with cystic fibrosis. Analysis of the cellular composition of airway and lung organoids showed that airway organoids contain proximal lung epithelial cells, while lung organoids contain both proximal and distal lung epithelial cells. Forskolin-induced swelling of organoids derived from a healthy donor showed that lung organoids, as well as airway organoids, contain functional epithelial cells and swell after 24 h exposure to forskolin, which makes it a suitable model for analyzing the cystic fibrosis transmembrane conductance regulator (CFTR) channel conductance in vitro. Thus, our results demonstrate the feasibility of generating and characterizing airway and lung organoids from hiPSCs, which can be used for a variety of future applications.

## 1. Introduction

The development of models for studying complex respiratory diseases in vitro and screening for drugs against a variety of genetic and respiratory diseases is an important goal of cell biology. Organoids have proven to be very valuable for these purposes. Organoids are three-dimensional (3D) structures derived from either pluripotent stem cells (PSCs), neonatal tissue stem cells, or adult stem cells (AdSCs)/adult progenitors, in which cells spontaneously self-organize into properly differentiated functional cell types and progenitors; they resemble their in vivo counterparts and model at least some function of the organ [1]. Organoids are used for disease modeling, drug screening, regenerative medicine, and for studying intercellular interactions [2]. In particular, airway and lung organoids are used for modeling lung disease [3,4] and personalized drug screening [5]. Cell-based regenerative therapy is considered to be a promising branch of regenerative medicine. In vivo studies on transplantation of lung organoids into injured lungs are currently on the rise, and some of them have shown positive regeneration of injured tissue after organoid implantation [6,7].

Human-induced PSCs (hiPSCs) are a suitable source of various types of organoids since obtaining somatic cells (peripheral blood monocytes or skin fibroblasts) for subsequent reprogramming is often a minimally invasive procedure, in contrast to obtaining tissue-specific cells. Due to their pluripotency, hiPSCs are universal; therefore, they can be used to obtain different types of organoids from the same patient, which is important for the study and treatment of hereditary multisystem diseases. There are many protocols for obtaining airway and lung organoids from hiPSCs [6,7,8,9,10,11], but they need to be improved and optimized to simplify the procedure.

A number of human monogenic hereditary diseases are associated with disruption of ion channels, for example, cystic fibrosis, which occurs due to mutations in the cystic fibrosis transmembrane conductance regulator (*CFTR)* gene encoding a chloride ion channel on the apical surface of epithelial cells [12]. Different types of mutations in the *CFTR* gene affect the protein in different ways, but all of them are characterized by a decrease in the channel function and, consequently, a disruption in the transport of chloride and sodium ions [13]. In 2013, Dekkers et al. suggested using forskolin-induced swelling (FIS) for functional analysis of the CFTR channel conductance in intestinal organoids [14]. The test allows for evaluating the functioning of the channel in normally cultivated organoids as well as in organoids treated with various drugs developed for therapy. The organoids used in this test are grown from the biopsy samples taken from the rectum of patients. Although the procedure is rather invasive, this test has recently entered the clinical practice in many countries to evaluate the effectiveness of therapy with CFTR modulators [15,16,17,18].

Previously published work [11] demonstrated that airway organoids derived from induced PSCs (iPSCs) can also be used to assess the CFTR channel conductance in the FIS assay. However, the applicability of lung organoids for the same purpose has not yet been investigated.

In this article, we describe the differentiation, cultivation, and cryopreservation of airway and lung organoids (AOs and LOs, respectively) from hiPSCs and characterize their cellular composition. We also demonstrate that both types of organoids can be used for functional assessment of the CFTR channel by the FIS assay, using the organoids from a healthy donor (*wt/wt*) and from two cystic fibrosis patients with homozygous F508del mutation of the *CFTR* gene (F508del/F508del).

## 2. Results

### 2.1. Differentiation of hiPSCs into Airway and Lung Organoids

**Figure 1:**
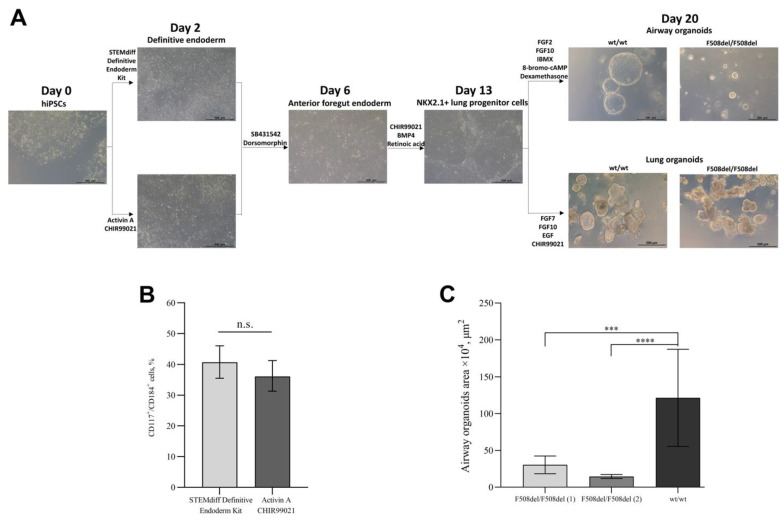
(**A**) Representative phase-contrast images of each stage of differentiation of human-induced PSCs (hiPSCs) into airway and lung organoids. Scale bar, 500 μm. (**B**) Flow cytometry assessment of CD117 and CD184 expression in definitive endoderm cells obtained by two protocols. Data are presented as mean value ± 95% CI, *n* = 3 biological replicates from independent differentiation wells, n.s.—not significant. (**C**) Quantification of the area of airway organoids derived from two cultures of F508del/F508del hiPSC and *wt/wt* hiPSC. Data are presented as mean value ± 95% CI, *n* = 3 technical replicates ***—*p* < 0.001, ****—*p* < 0.0001.

We differentiated hiPSCs lines into airway and lung organoids. Briefly, hiPSCs were differentiated into definitive endoderm, followed by the formation of anterior foregut endoderm and subsequently NKX2.1+ lung progenitor cells, which were finally differentiated into airway or lung organoids. There are different protocols of differentiation of hiPSCs into definitive endoderm cells, we chose two protocols: (a) using the STEMdiff Definitive Endoderm Kit or (b) using the Activin A and CHIR99021. There were no morphological differences between the cells obtained by these two protocols (Figure 1A). The percentage of cells with the co-expression of the surface markers of definitive endoderm CD117 and CD184 after 72 h of differentiation was 40.7% (95% CI: 35.5–46, *n* = 3) for the STEMdiff Definitive Endoderm Kit protocol and 36.1% (95% CI: 31.3–41.3, *n* = 3) for the Activin A and CHIR99021 protocol, with no statistically significant differences (*p* = 0.2) (Figure 1B). Based on these results, we conclude that both protocols differentiate hiPSCs into DE cells with equal efficiency. Then, we carried out the differentiation of definitive endoderm (DE) cells into anterior foregut endoderm (AFE) cells and AFE cells into NKX2.1+ lung progenitors. The percentage of CD47+/CD26- NKX2.1+ lung progenitors was 10.9 ± 7.2% (SD: 7.2, *n* = 18). NKX2.1+ cells on days 14–16 from the start of differentiation were resuspended in undiluted cold Matrigel with airway or lung medium. The formation of organoids was observed on days 1–2.

At each stage of differentiation (DE, AFE, NKX2.1+ lung progenitor cells, AOs, and LOs), the expression of specific markers was assessed by qRT-PCR (Figure S1). As expected, gene expression of most of the markers (*SOX17* and *GATA6*, definitive endoderm markers; *FOXA2* and *TBX1*, anterior foregut endoderm markers; *NKX2.1* and *SOX9*, lung lineage markers; *SCGB3A2*, airway organoids marker; *AQP1* and *SFTPC*, lung organoids markers; primer sequences are found in Table S1) was increased compared to undifferentiated hiPSCs, with the exception of *FOXJ1* (a marker of the proximal airway cells), which was decreased.

The sizes of AOs derived from *wt/wt* hiPSCs, and F508del/F508del hiPSCs are different (Figure 1A). We assessed the areas of airway organoids derived from *wt/wt* hiPSCs and two lines of organoids derived from F508del/F508del hiPSCs (Figure 1C) and showed that the average areas of *wt/wt* and F508del/F508del AOs were 121.7 mm^2^ (95% CI: 76–167.3) and 21.4 mm^2^ (95% CI: 10–30.9), respectively.

### 2.2. Cellular Composition of Airway and Lung Organoids

**Figure 2:**
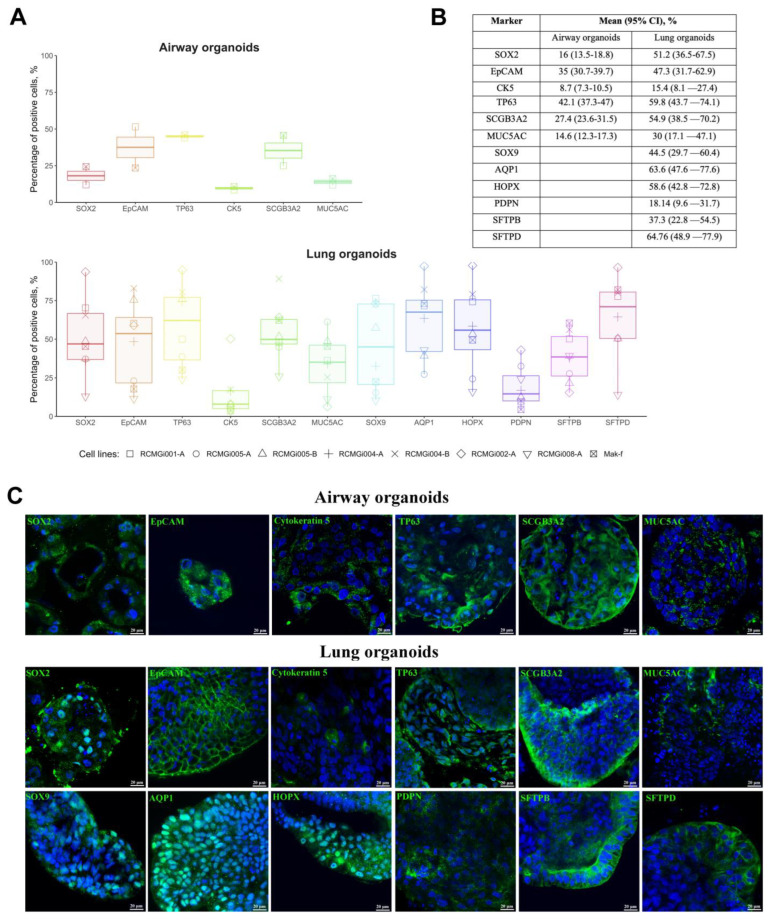
(**A**,**B**) Proportion of cells expressing specific markers in airway and lung organoids (AOs and LOs, respectively) at 22–31 days (median, 25th–75th percentile). (**A**)—Cytometry, (**B**)—confocal microscopy. (**C**) Representative images from confocal microscopy of AOs and LOs stained against major lung epithelial cell markers at 22–31 days. Nuclei were stained with DAPI (blue). Scale bar, 20 μm.

To use organoids for modeling lung diseases, developing new methods of treatment and diagnostics, it was necessary to determine the cellular composition of airway and lung organoids. AOs and LOs were derived from one cell line generated from healthy donor and seven cell lines generated from five cystic fibrosis patients. The cellular composition of the organoids was determined by the analysis of major lung epithelial cell markers by flow cytometry (Figure 2A,B) and confocal microscopy (Figure 2C). We demonstrate that AOs contain the proximal lung epithelial progenitor cells (SOX2+ cells, EpCAM+ cells), basal (Cytokeratin 5+ (CK5+) cells, TP63+ cells), Club (SCGB3A2+ cells), and goblet (MUC5AC+ cells) cells, and LOs contain the same types of cells plus distal lung epithelial progenitor cells (SOX9+ cells), which include alveolar type I (AQP1+ cells, HOPX+ cells) and type II (SFTPB+ cells, SFTPD+ cells) cells.

Surprisingly, we found that SOX2 and TP63 are expressed in the cytoplasm of the airway organoid cells, while they are expected to have a nuclear localization. In an attempt to understand this phenomenon, we performed immunostaining of intestinal organoids derived from a rectal biopsy of a cystic fibrosis patient. They demonstrated the same pattern of distribution of these markers in the cytoplasm (Figure S2).

We assumed that at earlier stages of organoid differentiation, these markers have a nuclear localization and ensure the maintenance of the multipotent status. However, in the process of differentiation (that is, maturation of the organoids), these proteins move into the cytoplasm. Therefore, we also immunostained for the SOX2 marker at days 3, 6, and 14 of hiPSCs differentiation into airway or lung organoids (Figure S3). According to the results of immunostaining, on days 3 and 6 of differentiation, SOX2 had a nuclear localization, while on day 14 of differentiation, part of SOX2 had cytoplasmic localization.

### 2.3. Forskolin-Induced Swelling of Organoids

**Figure 3:**
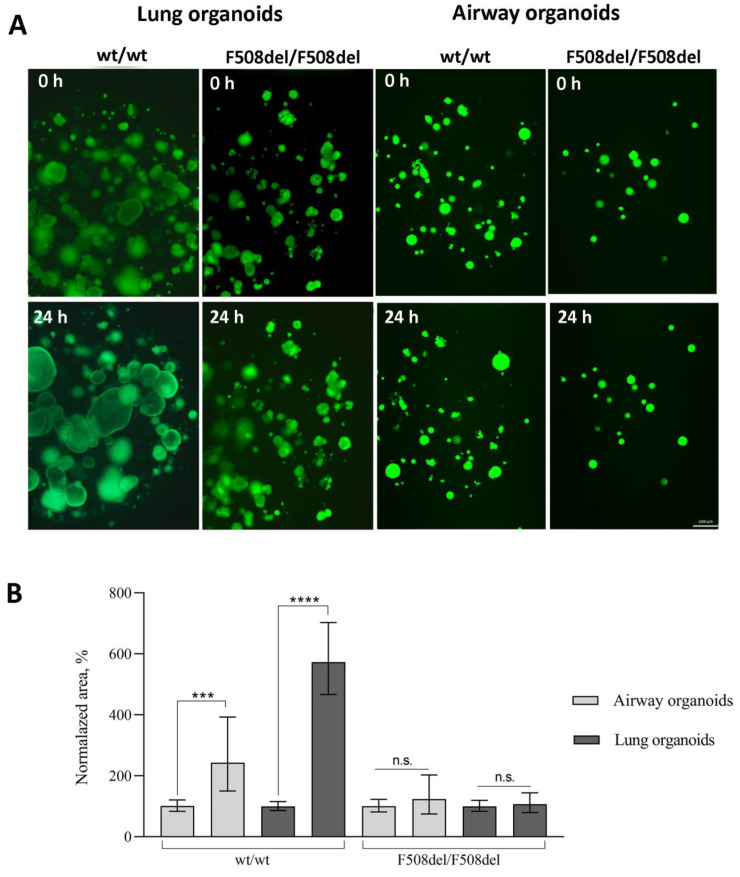
Forskolin-induced swelling of airway and lung organoids. (**A**) Representative images of F508del/F508del or *wt/wt* airway and lung organoids before and after 24 h of stimulation with forskolin (10 µM). Scale bar = 1000 µm. (**B**) Quantification of the normalized (by time point 0) swelling area of airway and lung organoids derived from F508del/F508del hiPSCs or *wt/wt* hiPSCs at time = 0 and 24 h. Data are presented as the mean ± 95% CI, *n* = 2 biological replicates from independent experiments. The number of analyzed organoids in each group is 30–150 individual organoids. ***—*p* < 0.001, ****—*p* < 0.0001, n.s.—not significant.

For functional analysis of the conductivity of the CFTR channel in organoids, an FIS assay of AOs and LOs was performed (Figure 3A). It has been previously reported that the activation of adenylyl cyclase by forskolin induces CFTR-dependent airway organoid swelling [11]. For the FIS assay, we reduced the final concentration of calcein green with respect to the protocol McCauley K. B. et al. [11]. This was due to the fact that at a concentration of calcein green (10 μM), we observed the death of organoids by 24 h of the analysis; in connection with this, the optimal selected concentrations for organoids in our work were 0.01 μM for airway organoids and 1 μM for lung organoids that is matched with the final concentration of calcein green for intestinal organoids [18]. AOs and LOs derived from *wt/wt* hiPSCs swelled by 24 h 2.4 times (95% CI: 1.4–4, *p* < 0.001) and 5.7 times (95% CI: 4.5–7.4, *p* < 0.0001), respectively, relative to the time point of 0 h. AOs and LOs derived from F508del/F508del hiPSCs swelled by 24 h 1.2 times (95% CI: 0.7–2.1, *p* = 0.4) and 1.1 times (95% CI: 0.8–1.5, *p* = 0.7), respectively, relative to the time point of 0 h (Figure 3B). The obtained results confirm the conclusion that LOs derived from hiPSC contain functional epithelial cells, the presence of which allows one to analyze the functionality of the CFTR channel in vitro.

We found that lung organoids swell more than airway organoids when supplemented with forskolin, which may be due to higher expression of CFTR in the LOs. We performed immunostaining on the CFTR of AOs, and LOs derived from a healthy donor and showed by flow cytometry that LOs express more CFTR proteins than AOs (Figure S4).

## 3. Discussion

Derivation of organoids from hiPSCs can be useful for many purposes, including disease modeling, drug screening, and evaluating the effectiveness of genome editing and regenerative therapy. Here we report a protocol for the derivation of airway and lung organoids from hiPSCs using a modified version of the protocols published by McCauley et al. [8] and Leibel et al. [9]. We performed differentiation of hiPSCs into definitive endoderm cells in two ways, using the STEMdiff Definitive Endoderm Kit or using Activin A and CHIR99021, and showed that the protocols do not differ in the efficiency of deriving cells. The anterior foregut endoderm differentiation from definitive endoderm cells was performed using SB431542 and dorsomorphin. After that, we performed differentiation of the cells into NKX2.1+ lung progenitors. In our work, the percentage of NKX2.1+ lung progenitors based on CD47+/CD26- was 10.9 ± 7.2%, in accordance with the results of a recent study by K. B. McCauley’s group, where the percentage of NKX2.1+ ranged from 14.3% to 21.9% [8,11]. It was shown previously that LOs could be obtained without sorting NKX2.1+ cells [9]; we have shown that it is possible to obtain AOs without also sorting, which simplifies the protocol of differentiation.

Gene expression analysis by qRT-PCR confirmed increased expression of the specific markers (*SOX17*, *GATA6*, *FOXA2*, *TBX1*, *NKX2.1*, *SOX9*, *SCGB3A2*, *AQP1,* and *SFTPC*) of each stage of differentiation (DE, AFE, NKX2.1+ lung progenitor cells, AOs, and LOs) compare to undifferentiated hiPSCs. However, we observed decreased expression of *FOXJ1* specific for AOs. FOXJ1 is a transcription factor that induces the growth of cilia [19]. It was also shown that *FOXJ1* expression is upregulated to induce and maintain iPSCs [20]. Since we performed expression analysis of the whole organoids, it is probable that the proportion of the ciliated cells in AOs is too low to reflect *FOXJ1* elevated expression compared to more homogeneous iPSCs culture. According to the results of the cellular analysis, AOs contain proximal lung epithelial cells (proximal lung epithelial progenitors, basal, Club, and goblet cells), while LOs contain both proximal and distal (alveolar type I and type II cells) lung epithelial cells. The localization of most of the markers we studied was typical, with the exception of SOX2 and TP63 in AOs. We investigated the localization of these markers in intestinal organoids derived from rectal biopsy, where we also found the cytoplasmic localization of these markers. According to the literature, in some cases, the cytoplasmic localization of SOX2 and TP63 may be associated with tumors [21,22]. In addition, the cytoplasmic localization of SOX2 may be associated either with acetylation at a lysine residue [23] or with mutations in the DNA-binding HMG-box domain of SOX2 [24]. Moreover, Yasuhara et al. showed in the example of Oct3/4 that nuclear import of Oct3/4 depends on transport factors, namely importin-α1/β [25].

In order to work with organoids, it was necessary to develop protocols for their passaging and cryopreservation. Replating organoids was performed every 1–2 weeks or as the organoids grew and/or “aged”. Existing protocols for passaging airway and lung organoids are different. According to McCauley et al. [8], airway organoids should be replated by an enzymatic method; however, we were unable to reproduce this method in our laboratory, which led to the death of airway organoids. Therefore, we carried out replating airway and lung organoids using a mechanical method without a syringe, according to Miller et al. [10].

We did not find any articles describing protocols for freezing airway organoids. Hawkins et al. described cryopreservation of airway basal cells derived from airway organoids but not airway organoids themselves [26]. We performed cryopreservation of lung organoids after their mechanical removal in the SFDM medium supplemented with 10% dimethyl sulfoxide and 10 μM Y-27632. Salahudeen et al. described cryopreservation of lung organoids enzymatically destructed to single cells; however, in our work, the survival of the cells after thawing was worse than the survival of the mechanically destructed organoids [27].

We demonstrated that the areas of airway organoids derived from *wt/wt* hiPSCs and F508del/F508del hiPSCs are statistically different due to the fact that airway organoids from a healthy donor have a large lumen, in contrast to airway organoids from cystic fibrosis patients. This phenomenon has been previously shown in intestinal organoids [28].

For several years, the FIS assay on intestinal organoids has been actively used to assess the effectiveness of CFTR modulators for rare *CFTR* mutations [15,29,30]. However, according to the Human Protein Atlas (https://www.proteinatlas.org/ENSG00000001626-CFTR/tissue, (accessed on 4 April 2022)), the levels of CFTR expression (both protein and mRNA) in the lungs and intestines are different. Higher levels of *CFTR* expression in the intestine may lead to false positive results of the FIS assay when evaluating the efficacy of therapy. In this regard, it is advisable to search for alternative models containing lung cells. In this study, we showed for the first time that lung organoids can be used to assess the functional activity of the CFTR channel using the FIS assay and confirmed that airway organoids could be used for the same purpose. Airway organoids from healthy hiPSCs and from hiPSCs with a cystic fibrosis mutation swelled after 24 h 2.4 and 1.2 times, respectively. Our results are consistent with the results of other studies, where healthy AOs swelled approximately 2.5 times, while AOs with cystic fibrosis mutation practically did not swell [8,31]. Lung organoids with a cystic fibrosis mutation also did not swell in response to forskolin, but healthy lung organoids swelled 5.7 times.

CFTR channel is expressed in many lung cells: secretory, basal, ciliated, alveolar cells, and ionocytes [32,33]. A stronger response to forskolin in lung organoids than in airway organoids may be due to the presence of a higher percentage of cells with CFTR channels in lung organoids, which is supported by our results.

To summarize, we described in detail enhanced and simplified protocols for derivation, cultivation, and cryopreservation of airway and lung organoids from hiPSCs, and characterized their cellular composition. We demonstrated for the first time that functionally active CFTR channels in healthy organoids can be successfully used in the forskolin test. The obtained airway and lung organoids may be used for a variety of future applications, such as lung disease modeling, personalized drug screening, and regenerative therapy.

## 4. Materials and Methods

### 4.1. Differentiation of hiPSCs into Airway and Lung Organoids

The study was approved by the Ethics Committee of the Research Centre for Medical Genetics (Moscow, Russia) and conducted in accordance with provisions of the Declaration of Helsinki of 1975. Patients and healthy donors signed informed written consent forms as anonymous participants of the study and donors of biological materials. Skin fibroblasts from a healthy donor (*wt/wt*) and five cystic fibrosis patients with homozygous or heterozygous F508del mutation of the *CFTR* gene were used for reprogramming. The derived hiPSC lines were previously described in the articles [34,35,36,37,38,39]. Skin fibroblasts and hiPSCs were deposited and are now available at the Moscow Branch of the Biobank “All-Russian Collection of Biological Samples of Hereditary Diseases” (Research Centre for Medical Genetics, Moscow, Russia).

Obtaining AOs and LOs from hiPSCs was carried out according to the protocols of McCauley et al. [8] and Leibel et al. [9], respectively, with modifications. Prior to differentiation, the hiPSCs lines were maintained under feeder-free conditions in the 6-well tissue culture dishes (Costar, Cambridge, MA, USA cat. no. 3506) coated with the Vitronectin (VTN-N) Recombinant Human Protein (Thermo Fisher Scientific, Waltham, MA, USA, cat. no. A14700) in the mTeSR1 medium (STEMCELL Technologies, Vancouver, BC, Canada, cat. no. 85850) for 14 days.

Differentiation of hiPSCs into airway and lung organoids consists of four stages. First, hiPSCs were differentiated into DE cells by two protocols: (a) using the STEMdiff Definitive Endoderm Kit (STEMCELL Technologies, cat. no. 05110) according to the manufacturer’s instructions or (b) using the Activin A and CHIR99021 [40]. On the first day of the differentiation, cell confluence for the protocol using the STEMdiff Definitive Endoderm Kit comprised 70–90%, whereas, for the protocol using Activin A and CHIR99021, it was 50–70%. The medium for differentiation using Activin A and CHIR99021 consisted of: RPMI-1640 (Paneco, Russia, cat. no. С330п) with 50× B-27 (Thermo Fisher Scientific, cat. no. 17504044), 100× GlutaMAX (Thermo Fisher Scientific, cat. no. 35050061), 100× penicillin-streptomycin (Paneco, cat. no. А063), 5 µM CHIR99021 (Tocris Bioscience, Bristol, U.K., cat. no. 4423) and 100 ng/mL human Activin A protein (R&D Systems, Minneapolis, MN, USA, cat. no. 338-AC-010). A total of 24 and 48 h after the start of differentiation using the Activin A and CHIR99021 protocol, the medium was replaced with the CHIR99021-free medium. A total of 72 h after the start of differentiation, cells were analyzed by flow cytometry for the efficiency of DE induction, which was assessed by co-expression of the surface markers of the definitive endoderm CD117 (Thermo Fisher Scientific, cat. no. 11-1178-41) and CD184 (Thermo Fisher Scientific, cat. no. 12-9999-41) according to the manufacturer’s protocol. Live cells were stained with Calcein Blue (Thermo Fisher Scientific, cat. no. C1429) and analyzed on a CytoFLEX S flow cytometer (Beckman Coulter, Brea, CA, USA) after filtering the cells through a 50 µm filter (Partec, Münster, Germany, cat. no. 04-0042-2317). Stained cells were stored at +4 °C in the dark until analysis.

The second stage was the differentiation of the DE cells into AFE cells. The cells were harvested with the Versene solution (PanEco, cat. no. Р080п) and passaged at a ratio of 1:3 if the Activin A and CHIR99021 protocol was used, and at a ratio of 1:4–1:5, if the STEMdiff Definitive Endoderm Kit protocol was used. The cells were passaged in 6-well tissue culture dishes coated with VTN-N in the serum-free differentiation medium (SFDM) supplemented with 10 μM SB431542 (Tocris, cat. no. 1614), 2 μM dorsomorphin (Tocris, cat. no. 3093), and 1 µM Y-27632 (STEMCELL Technologies, cat. no. 72302). The SFDM medium consists of 75% IMDM (Thermo Fisher Scientific, cat. no. 21980032), 25% Ham’s F12 (Thermo Fisher Scientific, cat. no. 11765054), 100Х B-27 (Thermo Fisher Scientific, cat. no. 17504044), 200Х N2 (Thermo Fisher Scientific, cat. no. 17502048), 0.05% bovine serum albumin solution (Sigma Aldrich, St. Louis, MO, USA, cat. no. A8412), 0.45 mM 1-thioglycerol (Sigma Aldrich, cat. no. M6145), 100Х GlutaMAX (Thermo Fisher Scientific, cat. no. 35050061), 0.05 mg/mL l-Ascorbic acid (Sigma Aldrich, cat. no. A4544) and 100 µg/mL primocin (InvivoGen, San Diego, CA, USA, cat. no ant-pm-2). After 24 and 48 h, the medium was replaced with the Y-27632-free medium.

The third stage was the differentiation of the AFE cells into NKX2.1+ lung progenitor cells. For this, the medium was changed to the SFDM medium supplemented with 3 µM CHIR99021, 10 ng/mL BMP4 (R&D Systems, cat. no. 314-BP-050), and 100 nM retinoic acid (Sigma Aldrich, cat. no. R2625). The medium was changed every other day for the next 8–10 days; just before the medium change, retinoic acid was added to the medium. At the end of this stage of differentiation, cells may be subjected to freezing, followed by their thawing and placing in Matrigel to form the organoids described below. The efficiency of differentiation into NKX2.1+ lung progenitor cells was analyzed by staining for CD47+/CD26- on days 14 from the start of differentiation. For this, cells were harvested with 0.05% trypsin-EDTA (PanEco, cat. no. П043п) and stained with anti-CD47 (Biolegend, San Diego, CA, USA, cat. no. 127529) and anti-CD26 (Biolegend, cat. no. 302705) antibodies according to the manufacturer’s protocols. The analysis was performed on a CytoFLEX S flow cytometer (Beckman Coulter). Before assessing the expression of markers, the cells were passed through a filter with a pore size of 50 μm. Stained cells were stored at +4 °C in the dark until analysis.

The last stage was the differentiation of the NKX2.1+ lung progenitor cells into airway and lung organoids on days 14–16 from the start of differentiation. To obtain airway and lung organoids from NKX2.1+ lung progenitor cells, cells were harvested with 0.05% trypsin-EDTA, counted using a Countess II FL Automated Cell Counter (Thermo Fisher Scientific), and centrifuged at 150× *g* for 5 min. The pellet was resuspended in undiluted cold Matrigel (BD Biosciences, San Jose, CA, USA, cat. no. 356234) at a concentration of 400–1000 cells/μL and replated in 20–50 μL drops into the wells of a 24-well plate (Corning, Somerville, MA, USA, cat. no. 3526). The drops were allowed to solidify for 40 min in an incubator, after which the medium for respiratory organoids supplemented with 10 μM Y-27632 was added. After 24 h, the medium was replaced with the Y-27632-free medium. The medium for airway organoids (airway medium) consisted of the SFDM medium supplemented with 250 ng/mL FGF2 (R&D Systems, cat. no. 233-FB), 100 ng/mL FGF10 (R&D Systems, cat. no. 345-FG), 50 nM dexamethasone (Sigma Aldrich, cat. no. D4902), 0.1 mM 8-bromo-cAMP (Sigma Aldrich, cat. no. I5879) and 0.1 mM 3-isobutyl-1-methylxanthine (Sigma Aldrich, cat. no. B5386). The medium for lung organoids (lung medium) consisted of the SFDM medium supplemented with 10 ng/mL FGF7 (R&D Systems, cat. no. 251-KG), 10 ng/mL FGF10, 10 ng/mL EGF (R&D Systems, cat. no. 2028-EG) and 3 µM CHIR99021.

Passaging of organoids was carried out according to Miller A. J. et al. [10]. The three-dimensional extracellular matrix with organoids was mechanically dislodged with a p1000 pipette tip precoated with 1% BSA (New England Biolabs Inc., Ipswich, MA, USA, cat. no. B9000 S) in DPBS without Ca^2+^ and Mg^2+^ (1% BSA-DPBS) to prevent adhesion of organoids to the tip. The dislodged droplets with organoids were placed into a 1.5 mL tube. Then organoids were passed 2–3 times through a syringe with a 27–31 g needle, precoated with 1% BSA-DPBS and centrifuged for 5 s at 6300× *g*. The pellet was resuspended in undiluted cold Matrigel at a concentration of 400–1000 cells/μL and replated in 20–50 μL drops into the wells of a 24-well plate. The drops were allowed to solidify for 40 min in an incubator after that medium for airway or lung organoids was added. The organoids were passaged once every 1–2 weeks or as the organoids grew and accumulated cellular debris within lumens or sank to the bottom of the Matrigel droplet.

For cryopreservation of lung organoids, droplets were mechanically dislodged, as described, and centrifuged for 5 s at 6300× *g*, the pellet was resuspended in SFDM medium with 10% dimethyl sulfoxide (Merck, Rahway, NJ, USA, cat. no. D2438) and 10 μM Y-27632, pre-wetted with a pipette tip in FACS buffer, and transferred into cryovials (Corning-Costar, cat. no. 430488). Cryovials with organoids were placed in a CoolCell LX rack (BioCision, Larkspur, CA, USA, cat. no. BCS-405G) and placed at −80 °C for 1 day, and then transferred into liquid nitrogen for long-term storage. Lung organoids were thawed by adding warm SFDM medium (37 °C) dropwise and centrifuged for 5 s at 6300× *g*. The pellet was resuspended in undiluted cold Matrigel at a concentration of 400–1000 cells/μL and replated in 20–50 μL drops into the wells of the plate, solidified for 40 min in an incubator, after which medium for lung organoids was added.

Assessment of the area of airway organoids was performed on day 32 from the start of differentiation; 4 days before the analysis, airway organoids were passaged according to the method described above. On the day of the analysis, we added calcein green (Thermo Fisher Scientific, cat. no. C34852) at a final concentration of 2.5 μM to the wells with organoids and incubated the plates for 40 min in an incubator. For each group, 14 to 38 organoids were analyzed using an Axio Vert A1 inverted microscope (ZEISS, Stuttgart, Germany). The images were analyzed using the CellProfiler software v.4.2.1 (Broad Institute of MIT and Harvard, Cambridge, MA, USA) [41], where a binary mask of the boundaries of airway organoids was obtained based on the fluorescence images of calcein in the green channel. The determination of primary objects and segmentation were performed; the area of the organoid was calculated as the sum of pixels in the primary object. Using the GraphPad Prism software v.9.1.1 (GraphPad Software Inc., San Diego, CA, USA), the area of organoids in pixels was recalculated to μm^2^ based on the analytical scale built into the ZEISS Axio Vert A1 microscope when images were acquired with a 10× objective.

### 4.2. Immunostaining of Organoids

Immunostaining of airway and lung organoids was performed according to the protocols of Dekkers et al. [42]. Briefly, droplets with organoids were mechanically dislodged on days 22–31 from the start of differentiation as described above, centrifuged for 5 s at 6300× *g*, and fixed in a chilled 4% formalin solution for 45 min at +4 °C. Then the organoids were permeabilized in a cold solution of 0.1% Tween 20 (Merck, cat. no. P9416) for 10 min at +4 °C and centrifuged for 5 min at 70× *g* at +4 °C; the precipitate was blocked with a cold solution of 0.1% Triton X-100 and 0.2% BSA in DPBS without Ca^2+^ and Mg^2+^ for 15 min at +4 °C. Then, a solution of primary antibodies was added, and the mixture was incubated overnight at +4 °C (Table 1). The organoids were washed twice with a solution of 0.1% Triton X-100 and 0.2% BSA in DPBS without Ca^2+^ and Mg^2+^ for 2 h at +4 °C. Then a solution of the secondary antibodies was added, and the mixture was incubated overnight at +4 °C (Table 1); the organoids were washed twice with a solution of 0.1% Triton X-100 and 0.2% BSA in DPBS without Ca^2+^ and Mg^2+^ for 2 h at +4 °C. After that, the organoids were stained with DAPI (Abcam, Cambridge, U.K., cat. no. Ab104139) for 10 min at room temperature and subsequently centrifuged for 5 s at 6300× *g*; the pellet was resuspended in a solution of 2.5 mM fructose (Sigma Aldrich, cat. no. F0127) in 60% glycerol (PanReac Applichem, Darmstadt, Germany, cat. no. 141339.1211) and incubated for 20 min at room temperature. The suspension was transferred onto a glass slide (Pyrex (Corning), Charleroi, PA, USA, cat. no. 2948) and covered with a cover glass (Pyrex (Corning), cat. no. 2975-225); microscopy was performed on a TCS SP8 confocal laser scanning microscope (Leica Microsystems, Wetzlar, Germany).

For the flow cytometry screening of the cellular composition of Aos and Los, the organoids, after immunostaining, were dissociated into single cells by treatment with 0.05% trypsin-EDTA for 10 min in an incubator. The cells were then centrifuged at 150× *g* for 5 min, and the pellet was resuspended in DPBS without Ca^2+^ and Mg^2+^. The analysis was performed on a CytoFLEX S flow cytometer. Before assessing the expression of markers, the cells were passed through a 50 μm filter. The number of analyzed events was 20,000. Stained cells were stored at +4 °C in the dark until analysis.

### 4.3. Forskolin-Induced Swelling of Organoids

Forskolin-induced swelling of organoids was performed on day 22 or later after the start of differentiation. The day before the analysis, the organoids were passaged as described above into the wells of a 24-, 48-, or 96-well plate with a droplet volume of 3 μL. The media for the cultivation of organoids included the SFDM medium supplemented according to each type of organoids; however, 8-bromo-cAMP and 3-isobutyl-1-methylxanthine were absent in the AO medium for at least one day before the start of the analysis. On the day of the analysis, calcein green was added to the wells with organoids at a final concentration of 0.01 μM for airway organoids and 1 μM for lung organoids, and the mixture was incubated for 40 min in an incubator. An image was taken on the ZEISS Axio Vert A1 inverted microscope. After that, forskolin (Sigma-Aldrich, cat. no. F6886) was added at a final concentration of 10 μM, the mixture was incubated for 24 h, and an image was taken.

The resulting Images were analyzed using the ilastik v.1.3.3 (European Molecular Biology Laboratory) [43] and CellProfiler software v.4.2.1 [41]. Organoid boundaries were determined by machine learning based on fluorescent images of calcein in the green channel using the ilastik program. Based on the obtained probability masks, a binary mask of organoid boundaries was obtained using the CellProfiler program. The determination of primary objects and segmentation were performed; the area of the organoid was calculated as the sum of pixels in the primary object. Using the GraphPad Prism program, the area of organoids was normalized, where the average area of organoids in the group at 0 h after the addition of forskolin was taken as 100%.

### 4.4. Statistical Data Analysis

Statistical analysis of data was performed by GraphPad Prism v.9.1.1 and R v.4.1.2 to fit GLMM models and check model assumptions lme4 v.1.1-27.1, performance v.0.8 libraries were used. For descriptive statistics, gaussian, binomial, beta, or gamma mean with 95% CI were calculated. To compare the percentage of positive cells obtained by flow cytometry, beta GLMM models were fitted with random intercept. To compare the area of airway organoids or swelling areas organoids over time, gamma GLMM models were fitted with a random slope and intercept and post hoc comparisons by Tukey’s method. Data were considered significant if the *p*-value was <0.05.

## Figures and Tables

**Table 1:** Antibodies used in immunostaining of organoids.

Antibody	Concentration
SOX2 (Abcam, cat. no. ab79351)	10 μg/mL
EpCAM (Abcam, cat. no. ab20160)	40 μg/mL
Cytokeratin 5 (Abcam, cat. no. ab52635)	6 μg/mL
TP63 (Thermo Fisher Scientific, cat. no. 703809)	10 μg/mL
SCGB3A2 (Abcam, cat. no. ab181853)	26 μg/mL
MUC5AC (Thermo Fisher Scientific, cat. no. MA5-12178)	4 μg/mL
SOX9 (Thermo Fisher Scientific, cat. no. MA5-17177)	20 μg/mL
AQP1 (Sigma-Aldrich, cat. no. HPA019206)	1.5 μg/mL
HOPX (Thermo Fisher Scientific, cat. no. PA5-90538)	25.4 μg/mL
SFTPB (Thermo Fisher Scientific, cat. no. MA1-204)	20 μg/mL
SFTPD (Thermo Fisher Scientific, cat. no. PA5-115988)	20 μg/mL
PDPN (Thermo Fisher Scientific, cat. no. MA5-16267)	5 μg/mL
Goat Anti-Rabbit IgG H&L (Alexa Fluor 488) (Abcam, cat. no. ab150077)	20 μg/mL
Goat Anti-Mouse IgG H&L (Alexa Fluor 488) (Abcam, cat. no. ab150113)	20 μg/mL
Alexa Fluor 594 Goat Anti-Rat IgG H&L (Thermo Fisher Scientific, cat. no. A11007)	20 μg/mL

## Data Availability

The data presented in this study are available upon request from the corresponding author.

## Supplementary Materials

The supporting information can be downloaded at: https://www.mdpi.com/article/10.3390/ijms24076293/s1.
