# Supplementary material for: Airway and Lung Organoids from Human-Induced Pluripotent Stem Cells Can Be Used to Assess CFTR Conductance
Source: Int J Mol Sci. 2023 Mar 27;24(7):6293. doi: 10.3390/ijms24076293 (PMC10094586; doi:10.3390/ijms24076293)
Supplement: Supplementary file 1 [file ijms-24-06293-s001.zip › ijms-2241994-supplementary.pdf]

Supplementary results

qRT–PCR analysis of differentiation

Stages of differentiation (DE, AFE, NKX2.1+ lung progenitor cells, AOs and LOs) were confirmed by qRT-PCR analysis of the expression of specific markers (*SOX17*, *GATA6*, *FOXA2*, *TBX1*, *NKX2.1*, *SOX9*, *SCGB3A2*, *FOXJ1*, *AQP1* and *SFTPC*) (Figure S1). Expression of almost all specific markers, except FOXJ1, was increased compared to undifferentiated hiPSCs. Expression of *FOXJ1* (marker of the proximal airway cells) was decreased.

**Cell type** / **Gene**	**log2 Fold Change from hiPSCs (95% CI)**
**DE**	
SOX17	5.75 (5.23 — 6.26)
GATA6	6.63 (6.08 — 7.18)
**AFE**	
FOXA2	6.75 (6.27 — 7.24)
TBX1	7.63 (7.12 — 8.15)
**NKX2.1**	
NKX2.1	2.25 (1.48 — 3.03)
SOX9	0.38 (-0.77 — 1.54)
**AO**	
SCGB2A	2.34 (2.13 — 2.54)
FOXJ1	-8.16 (-7.36 — -8.96)
**LO**	
AQP1	1.54 (0.74 — 2.34)
SFTPC	2.13 (1.22 — 3.05)

Figure S1. Relative gene expression of various cell-type markers at each stage of differentiation of hiPSCs into AOs and LOs. Gene expression was normalized to the housekeeping genes (*B2M*, *TBX* and *TFRC*) and is represented in the table as log2 fold change relative to the expression levels in hiPSCs (expression level = 1.0), n = 2 biological replicates. DE – definitive endoderm, AFE - anterior foregut endoderm, NKX2.1 – NKX2.1-positive lung progenitor cells, AO – airway organoids, LO – lung organoids.

Immunostaining of intestinal organoids

We analyzed localization of the SOX2 and TP63 markers in intestinal organoids derived from human rectal biopsies. All of these markers, as well as airway organoids, had a cytoplasmic localization (Figure S2).

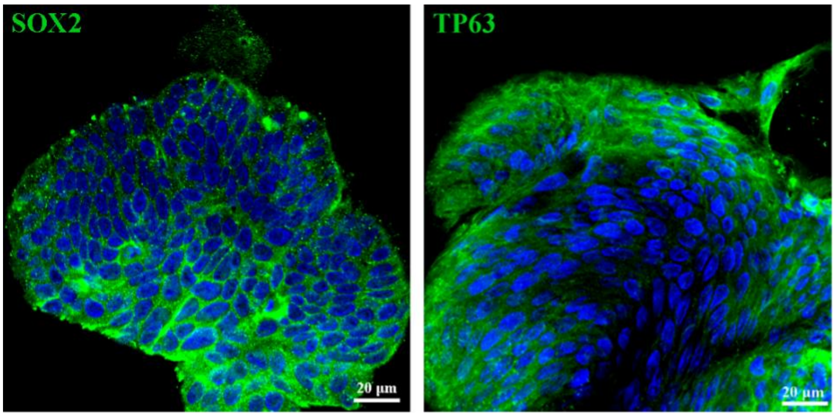

Figure S2. Confocal images of SOX2 and TP63 markers in intestinal organoids at day 7. Nuclei were stained with DAPI (blue). Scale bar, 20 µm.

Immunostaining of cells during differentiation

To analyze the localization of SOX2, we performed immunostaining of hiPSCs during differentiation into organoids. We chose three time points: day 3 (DE cells), day 6 (AFE cells) and day 14 (NKX2.1+ lung progenitor cells) from the start of differentiation. According to the results, the nuclear localization of SOX2 is maintained at the stages of obtaining DE and AFE cells, while NKX2.1+ lung progenitor cells have both cytoplasmic and nuclear localization of SOX2 (Figure S3).

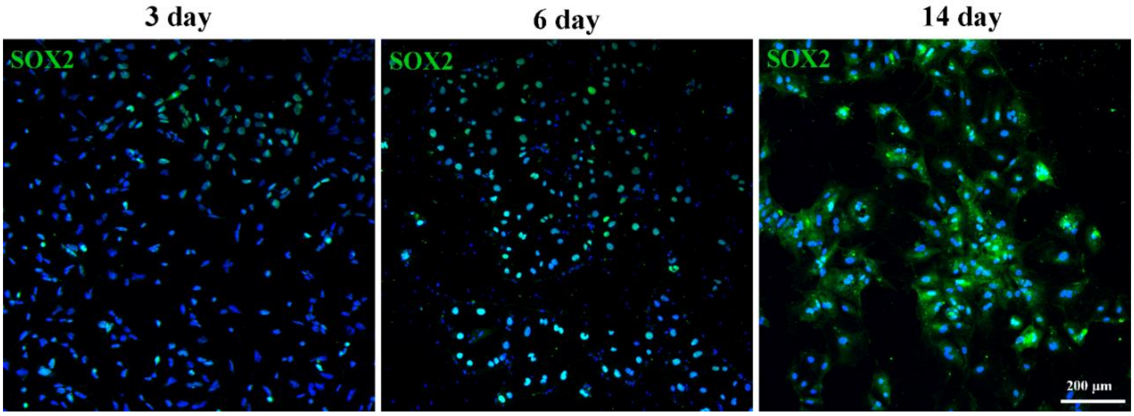

Figure S3. Fluorescent images of SOX2 markers in organoids at days 3 (DE cells), 6 (AFE cells) and 14 (NKX2.1+ lung progenitor cells) of differentiation. Nuclei were stained with DAPI (blue). Scale bar, 200 µm.

CFTR protein expression in airway and lung organoids

To confirm the presence of the CFTR channel in organoid cells, we performed a quantitative and qualitative assessment of the CFTR marker expression in airway and lung organoids derived from healthy hiPSCs at day 22 of differentiation. By immunostaining for

the CFTR marker, we confirmed the presence of CFTR channels in airway and lung organoid cells (Figure S4). The quantification of CFTR+ cells in AOs and LOs by flow cytometry comprised 13.7% and 75.3%, respectively. This result may indicate that LOs contain a higher percentage of cells expressing the CFTR channel then AOs; as a result, LOs respond to forskolin much stronger than AOs. However, current evidence is limited and further research is required.

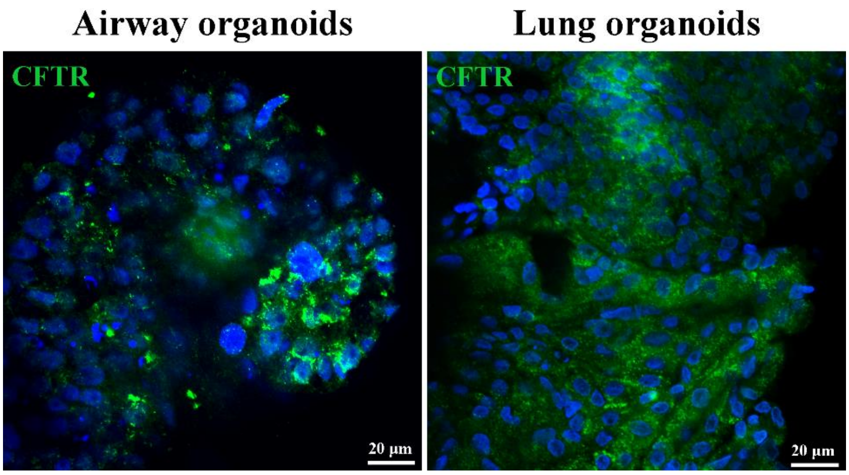

Figure S4. Confocal images of the CFTR marker in airway and lung organoids derived from a healthy donor at day 22 of differentiation. Nuclei were stained with DAPI (blue). Scale bar, 200 μm.

Supplementary materials and methods

RNA preparation and qRT–PCR

Total RNA was isolated from hiPSCs, DE cells, AFE cells, NKX2.1+ lung progenitor cells, AOs and LOs by using RNeasy kit (QIAGEN). cDNA was synthesized from 500 ng of total RNA using MMLV Reverse Transcriptase (Evrogen) according to the manufacturer protocol. 2 μL of cDNA was added to each 25 μL qPCR reaction. qPCR was performed in triplicate using the gene-specific primers (Table S1), SYBR green and Applied Biosystems QuantStudio 5 Real-Time PCR System. Cycling parameters were as follows: 95°C for 5 min, followed by 40 cycles of 20 s at 95°C, 30 s at 58°C and 25 s at 72°C. Relative gene expression was quantified using the ΔΔCt method, normalized by housekeeping genes (B2M, TBX and TFRC) and calculated as fold change.

Table S1. List of qRT–PCR primer sequences

Target	Forward/Reverse primer (5′-3′)
FOXA2	GCACTCGGCTTCCAGTATG

	TGTACGTGTTCATGCCGTTC
TBX1	GGACGACAACGGCCACATTA<br>GGTTCTGGTAGGCAGTGACC
SOX9	TGCCTTTGCTTGTTCACTGC<br>CTGAGAGGCACAGGTGACAG
NKX2.1	CACCTTGCTATACGGTCGGA<br>TCCTTGGTCTAAACGCGGC
SOX17	CGCTTTCATGGTGTGGGCTAAGGACG<br>TAGTTGGGGTGGTCCTGCATGTGCTG
GATA6	ACTTCCCCCACAACACAACC<br>GAGCCCATCTTGACCCGAAT
SCGB3A2	CAAGTGGAACCACTGGCTTG<br>CCAGAGGTAAAGGTGCCAAC
SFTPC	GTGAGCAGGGTCAGTGGAA<br>ACTCCCATCCTCTCCCCAAG
AQP1	GGGAAGAGATCCCAGGAGGT<br>CGATTCCAAGGTCAGGCAGT
B2M	CTGCCGTGTGAACCATGTGA<br>CAATCCAAATGCGGCATCTTC
TBP	CGGAGAGTTCTGGGATTGTAC<br>GTGGTTCGTGGCTCTCTTATC
TFRC	TCCTTGCATATTCTGGAATCCC<br>ATCACGAACTGACCAGCG

Derivation of intestinal organoids from human rectal biopsies

The protocols we employed were originally developed by the J.M. Beekman's team who used them for generation of intestinal organoids [17,18]. The study was approved by the Ethics Committee of the Research Centre for Medical Genetics (Moscow, Russia) and conducted in accordance with the provisions of the Declaration of Helsinki (1975). Patients with homozygous F508del mutation of the *CFTR* gene signed informed written consent forms as anonymous participants of the study and donors of biological materials.

The L-Wnt3A and Noggin-HEK293 cell lines were the courtesy of J.M. Beekman and were used to produce conditioned media with appropriate growth factors. Both cell lines

were maintained in the DMEM + GlutaMax medium (Thermo Fisher Scientific, cat. no. 10566016) supplemented with 10% Fetal Bovine Serum (PAA Laboratories, cat. no. A15101) and penicillin/streptomycin. For selective propagation of transfected L cells and HEK293 cells, the medium was supplemented with zeocin (Thermo Fisher Scientific, cat. no. R25001) at a concentration of 125 μg/ml or G418 (Thermo Fisher Scientific, cat. no. 10131035) at a concentration of 500 μg/ml, respectively. The conditioned media were obtained after 8–9 days of cultivation in the medium without selective antibiotics. The medium for culturing organoids contained 50% Wnt-3A- and 10% Noggin-conditioned media, 40% Advanced DMEM/F12 (Thermo Fisher Scientific, cat. no. 12634010), 50 ng/mL hEGF (Prospec, cat. no. CYT 217), 300 ng/ml hR-Spondin-3 (Peprotech, cat. no. 120-44), 2% B27, 1.25 mM N-acetylcysteine (Sigma-Aldrich, cat. no. A7250), 10 mM nicotinamide (Sigma-Aldrich, cat. no. N3376), 5 μM A83-01 (Tocris, cat. no. 29-391-0), 10 μM SB 202190, 100 μg/ml primocin [17, 18].

Crypt isolation from rectal biopsies was preceded by a series of washes with the Advanced DMEM/F12 medium and PBS solution. The biopsy samples were then incubated with a 10 mM EDTA solution (Thermo Fisher Scientific, cat. no. AM9260G) in PBS for 40 min with constant stirring at +4 °C. Upon completion of the incubation, the samples were resuspended to release individual crypts from the rectal biopsies. The resulting crypt suspension was precipitated by centrifugation for 5 min at 130 g and +4 °C. The crypt precipitate was mixed with Matrigel and then plated onto culture plates; these steps were performed on ice. The plates were placed for 30 min in a CO2 incubator to allow Matrigel polymerization with the embedded crypts. Then the growth medium was added to each well. The organoids were re-plated once a week. Prior to that, the growth medium was removed from all wells, Matrigel droplets were mechanically destroyed, and organoids were mechanically dissociated into smaller fragments. The resulting suspension was precipitated for 5 min at 130 g and +4 °C. The organoid precipitate was mixed with Matrigel and plated. The medium for crypts and organoids was replaced every 2–3 days.

Immunostaining assay

Immunostaining of intestinal, airway and lung organoids was carried out according to the protocols of Dekkers et al. [42].

For immunostaining of cells on days 3, 6 and 14 of differentiation, cells were fixed in a chilled 4% formalin solution for 15 min at +4 °C. Then cells were permeabilized in a solution of 0.1% Tween 20 in DPBS without Ca2+ and Mg2+ for 10 min at RT and blocked with a solution of 0.1% Tween 20 and 1% BSA in DPBS without Ca2+ and Mg2+ for 30 min at RT.

After that, a solution of SOX2 primary antibodies at a final concentration of 5 μg/ml was added and the mixture was incubated for 1 hour at RT, then rinsed three times with DPBS without Ca2+ and Mg2+. A solution of secondary antibody at a final concentration of 10 μg/ml was added and the mixture was incubated overnight at +4 °C, then rinsed three times with DPBS without Ca2+ and Mg2+. DAPI was added and the mixture was incubated for 10 min at RT. Microscopy was performed on a TCS SP8 confocal laser scanning microscope (Leica Microsystems).

Immunostaining for the CFTR marker was carried out in airway and lung organoids derived from a healthy donor at day 22 of differentiation. Primary Anti-CFTR antibody (Abcam, cat. no. ab131553) was used at a final concentration of 6 μg/ml. Secondary Goat Anti-Rabbit IgG H&L (Alexa Fluor 488) (Abcam, cat. no. ab150077) was used at a final concentration of 20 μg/ml. For the flow cytometry screening of cellular composition of AOs and LOs, organoids after immunostaining were dissociated into single cells using 0.05% trypsin-EDTA for 10 min in an incubator. The cells were then centrifuged at 150 g for 5 min and the pellet was resuspended in DPBS without Ca2+ and Mg2+. The analysis was performed on a CytoFLEX S flow cytometer. Before assessing expression of the markers, the cells were passed through a 50 μm filter. The number of analyzed events was 20,000. Stained cells were stored at +4 °C in the dark until analysis.
